# Endobronchial ultrasound in real life: primary diagnosis and mediastinal staging of lung cancer in patients submitted to thoracic surgery

**DOI:** 10.1186/s12890-016-0264-7

**Published:** 2016-07-19

**Authors:** João Pedro Steinhauser Motta, Axel Tobias Kempa, Alexandre Pinto Cardoso, Marcos Eduardo Paschoal, Ronir Raggio Luiz, José Roberto Lapa e Silva, Franz Stanzel

**Affiliations:** Instituto de Doenças do Torax, Universidade Federal do Rio de Janeiro, Rua Professor Rodolpho Paulo Rocco, 255, 1 andar, sala 01D 58/60, Rio de Janeiro, RJ 21941-913 Brazil; Klinikum-Stuttgart, Kriegbergstrasse 60, 70174 Stuttgart, Germany; Instituto de Estudos em Saúde Coletiva, Universidade Federal do Rio de Janeiro, Avenida HorácioMacedo, lha do Fundão - Cidade Universitária, 21941-598 Rio de Janeiro, RJ Brazil; Lungenklinik Hemer, Theo-Funccius-Straße 1, 58675 Hemer, NW Germany

## Abstract

**Background:**

Since the first articles published for over 10 years ago, endobronchial ultrasound (EBUS) has gained a strong scientific backing and has been incorporated into routine medical practice in pulmonology and thoracic surgery centers. How is EBUS performing outside the scientific environment, as a diagnostic and mediastinal staging tool in a subset of patients that undergo thoracic surgery, is an interesting question.

**Methods:**

This study evaluated consecutive patients who, during the period from January 2010 to August 2012, were submitted to EBUS and later to thoracic surgery. The samples obtained by endobronchial ultrasound-guided transbronchial needle aspiration (EBUS-TBNA) were compared to surgical samples. The primary endpoint was the proportion of patients with a final diagnosis of non-small cell lung cancer (NSCLC) by EBUS-TBNA correctly subtyped. The secondary endpoint was the negative predictive value (NPV) of EBUS-TBNA for mediastinal staging of lung cancer.

**Results:**

Two hundred eighty seven patients were studied. Considering 84 patients with a final diagnosis of NSCLC by EBUS-TBNA, 79 % (CI 95 % 70.1–87.3) were correctly subclassified. The NPV of EBUS-TBNA for mediastinal staging was 89 % (IC 95 % 84.9–92.7). From a total of 21 false negative cases of mediastinal staging, 16 (76 %) did not undergo positron emission tomography-computed tomography (PET-CT) before the EBUS and in 15 (71 %) the affected lymph node chain was not punctured by EBUS-TBNA. Ten (47 %) patients had only lymph node metastases not directly accessible by the EBUS.

**Conclusions:**

Performed in hospital routine and in patients submitted to thoracic surgery, EBUS-TBNA proved to be a good tool for proper pathological diagnosis of lung cancer. The negative predictive value of 89 % for mediastinal staging of lung cancer is comparable to that reported in previous studies, but the relatively high number of 21 false negative cases points to the need for standardization of routine strategies before, during and after EBUS.

## Background

Lung cancer is the malignant tumor with the highest mortality rate among men and women worldwide, with more than 1,4 million deaths a year [1]. The emergence of endobronchial ultrasound-guided transbronchial needle aspiration (EBUS-TBNA), a minimally invasive technique able to provide valuable information for a primary tumor diagnosis and mediastinal staging, significantly changed the approach to lung cancer [2, 3]. Since the first articles published for over 10 years ago [4], endobronchial ultrasound (EBUS) has gained strong scientific backing [5–7] and has been incorporated into routine medical practice of pulmonology and thoracic surgery centers. Guidelines of respiratory societies reinforce the importance of ultrasound-guided needle techniques in the primary diagnosis and mediastinal staging of lung cancer [8–11]. The success of the technique and the spread of its use around the world make it important to revaluate its performance in “real life”, outside the scientific environment, especially in patients undergoing thoracic surgery, in which the sharpest staging tools are required.

The aim of our study is to determine EBUS performance in patients undergoing thoracic surgery in hospital routine. Using surgical pathology as the gold standard, we calculated the proportion of patients with a final diagnosis of non-small cell lung cancer (NSCLC) by EBUS-TBNA correctly subtyped and the performance of EBUS-TBNA for the mediastinal staging of lung cancer.

## Methods

### Study design

The present work is a single-center, retrospective, observational study. All patients who underwent EBUS and thoracic surgery during the period from January 2010 to August 2012 were selected.

### Procedures

The examinations and surgeries took place in the Lungenklinik Hemer, a traditional center for respiratory diseases in Germany. EBUS procedures were performed on hospitalized patients, under general anesthesia. The examination could be accompanied by the presence of a pathologist in the procedure room for rapid on-site evaluation (ROSE) or not. EBUS-TBNA samples were collected using a 22-gauge needle. At least 2 aspirates were obtained from each target lesion. When a pathologist was present, 2 pairs of smears were prepared for ROSE and cytologic examinations. The resulting material (tissue cores, shreds of tissue, cellular components, fluids) was processed using a cell-block technique. If there was no pathologist in the room, all the samples were processed as cell-blocks. Indications for a surgical exploration or resection due to suspicion or confirmation of lung cancer were discussed and had to be approved by a local interdisciplinary clinical session with the mandatory participation of pulmonologists, oncologists, chest surgeons, radiologists and radiotherapists. EBUS-TBNA and surgical samples were analyzed by two different pathology services.

### Data collection

Data collection and statistical analysis were conducted at the Federal University of Rio de Janeiro, Brazil. Through the computer program Teamviewer ® (TeamViewer GmbH, Germany) the authors had remote access to a database provided by the Lungenklinik Hemer to access patient data from local electronic medical records.

### Inclusion criteria

Patients who underwent EBUS for any indication in the Lungenklinik Hemer from January 2010 to August 2012 and were subsequently subjected to surgical procedures.

### Exclusion criteria

Patients with no lymph node sampling by EBUS-TBNA during an EBUS procedure or patients who did not have histopathologic sampling of mediastinal lymph nodes during surgery.

### Study population

**Fig. 1:**
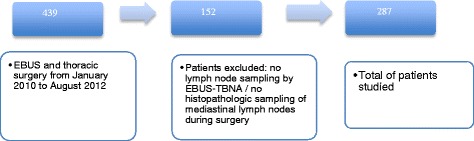
Flow chart showing selection, exclusion and total of patients studied. EBUS: endobronchial ultrasound. EBUS-TBNA: endobronchial ultrasound-guided transbronchial needle aspiration

Patients were identified in the electronic system of the Lungenklinik Hemer by crossing the specific code representing the EBUS with codes representing thoracic surgical procedures such as thoracotomy, thoracoscopy or mediastinoscopy. Patients whose electronic records showed both codes in the same hospital stay were selected. Four hundred thirty-nine patients underwent both an EBUS and thoracic surgery between January 2010 and August 2012 in the Lungenklinik Hemer. One hundred fifty-two cases were excluded for not having mediastinal lymph node sampling from both EBUS and surgery. The remaining 287 patients were studied (Fig. 1).

### Endpoints

The primary endpoint was the proportion of patients with a final diagnosis of NSCLC by EBUS-TBNA correctly subtyped as compared to the surgical samples. The secondary endpoint was the negative predictive value (NPV) of EBUS-TBNA for mediastinal staging of lung cancer, according to the 7^th^ edition of the lung cancer staging system of the American Joint Committee on Cancer [12].

### Statistical analysis

Patient demographics and disease characteristics were summarized using descriptive statistics. For the primary endpoint, the final result of the pathology after surgical resection was used as the gold standard for comparison to EBUS-TBNA samples. This result takes into account the tumor samples present in the lung parenchyma and/or mediastinum. There was no pairing of samples per lymph node site. For the secondary endpoint, the EBUS-TBNA samples were compared only to mediastinal surgical samples (obtained by mediastinoscopy and/or surgical mediastinal lymphadenectomy). The proportion of NSCLC correctly subclassified, the NPV for mediastinal staging and their 95 % confidence intervals (CI) were calculated using standard definitions. All statistical analyses were performed using SPSS® (IBM SPSS Statistics Version 20, United States of America).

## Results

**Fig. 2:**
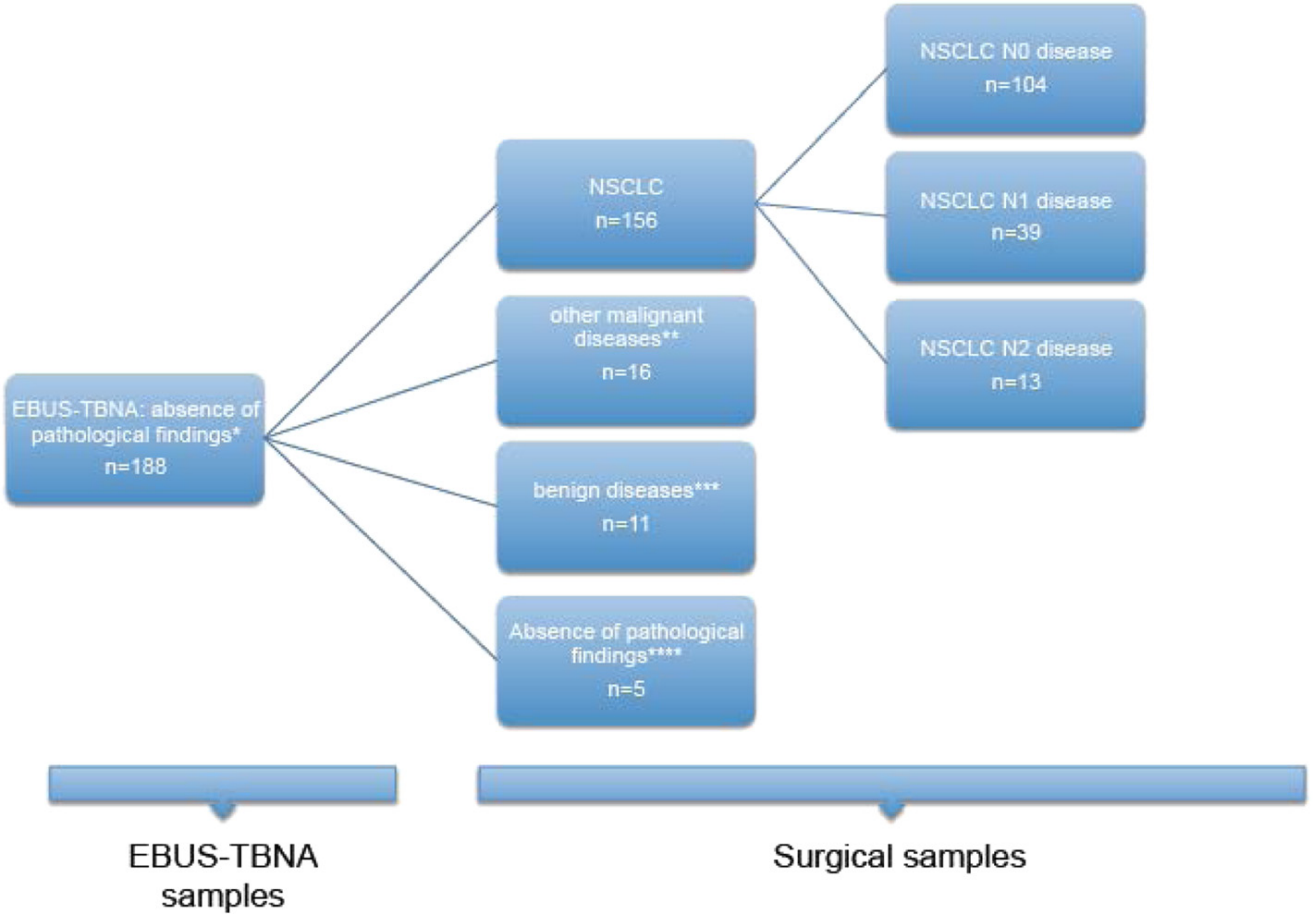
Flow chart showing the 188 cases with samples without pathological findings by EBUS-TBNA and the final pathological results after surgery. * EBUS-TBNA representative samples without pathological findings. ** surgical samples with malign pathological findings others than NSCLC. *** surgical samples with benign pathological findings. **** surgical samples without pathological findings

**Fig. 3:**
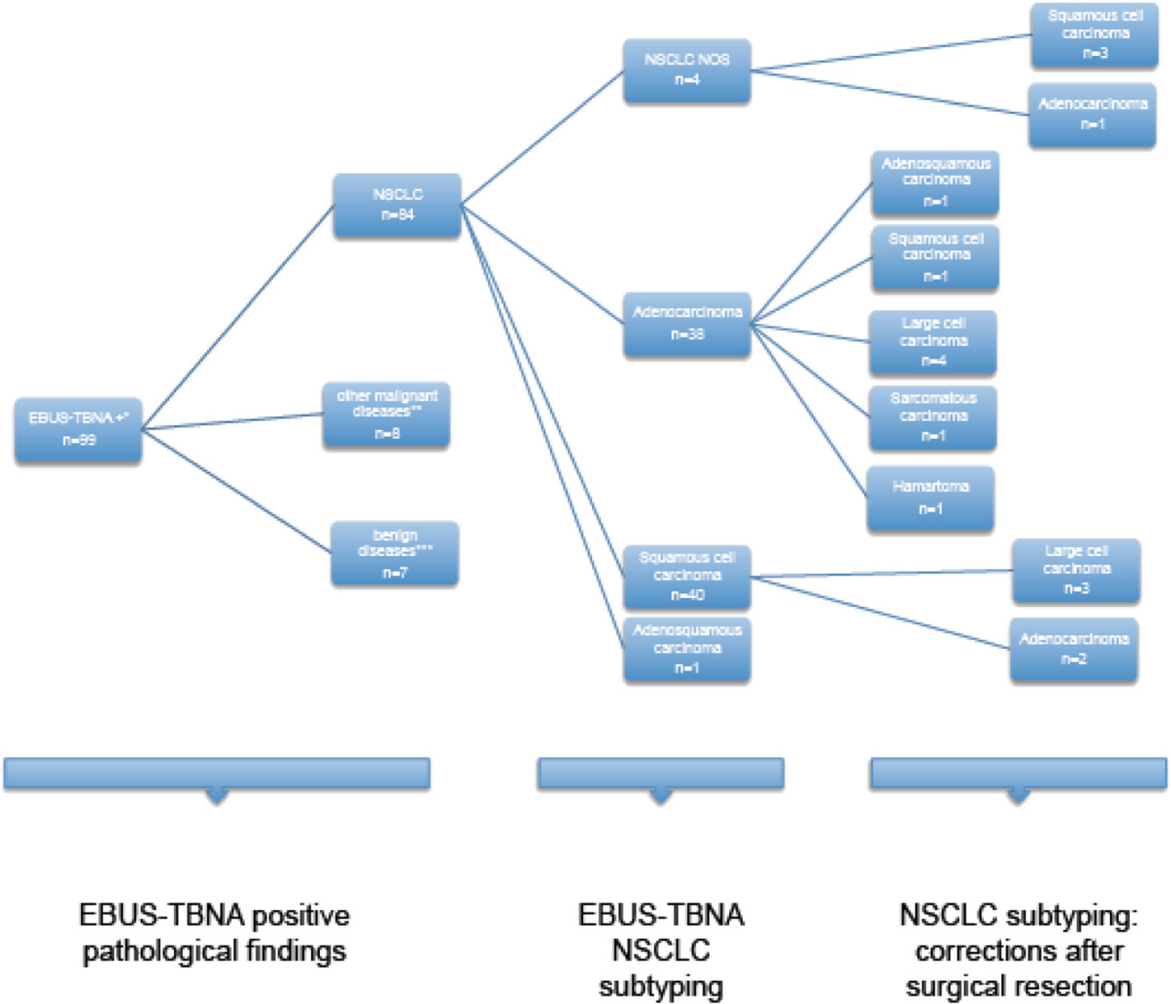
Flow chart showing pathological findings of the EBUS-TBNA samples; NSCLC subtyping by EBUS-TBNA and corrections after surgical resection. EBUS-TBNA: endobronchial ultrasound-guided transbronchial needle aspiration. NSCLC: non-small cell lung cancer. NSCLC NOS: non-small cell lung cancer not otherwise specified. * EBUS-TBNA samples with positive pathological findings. ** EBUS-TBNA samples with malign pathological findings others than NSCLC. ***EBUS-TBNA samples with benign pathological findings

The demographic data, tumor characteristics and details of the procedures performed are summarized in Table 1. The samples obtained by EBUS-TBNA showed no pathological findings in 188 patients (65.5 %) (representative lymph node samples without disease). NSCLC was detected in 84 patients (29.2 %), other malignant diseases in 8 (2.7 %) and benign pathological findings in 7 (2 %) patients (Table 2). Considering the 188 patients without pathological findings in samples of EBUS-TBNA, 156 had a final diagnosis of NSCLC after surgery. Of these, the final surgical mediastinal staging was N0 in 104 patients, N1 in 39 patients and N2 in 13 patients (Fig. 2). In 99 patients it was possible to compare the pathological findings of the EBUS-TBNA samples with the surgical findings. Taking into account NSCLC subtyping analysis, EBUS-TBNA was correct in 67 cases and incorrect or incomplete in 17 cases (Fig. 3). Four patients were diagnosed as NSCLC not otherwise specified (NSCLC-NOS) by EBUS and were determined to be squamous cell carcinoma (3 patients) or adenocarcinoma (1 patient) after the surgical resection; 1 was classified as pulmonary adenocarcinoma and determined to be adenosquamous carcinoma from the surgical sample; 7 were diagnosed as squamous cell carcinoma or adenocarcinoma and the surgical pathology showed large cell carcinoma; 2 cases were subtyped as squamous cell carcinoma and the surgical sample determined a diagnosis of adenocarcinoma; 1 case of adenocarcinoma by EBUS-TBNA was determined to be squamous cell carcinoma by the surgical sample; 1 case classified as adenocarcinoma by EBUS-TBNA and was determined as sarcomatous carcinoma by the surgical sample; and 1 single case of false malignancy of adenocarcinoma with the EBUS-TBNA samples, and surgical resection confirmed a diagnosis of hamartoma. Considering this subclassification, the proportion of patients with a final diagnosis of NSCLC by EBUS-TBNA in whom NSCLC subtyping was correct was 79 % (IC 95 % 70.1-87.3).

**Table 1:** Patient demographics, disease and procedure characteristics

Patients, N	287
Age, mean	64,7
Male gender, N (%)	204 (71.1)
Smoking History, N (%)	126 (43.9)^a^
COPD, N (%)	81 (28.2)
Cardiovascular Disease, N (%)	148 (51.6)
Cancer History, N (%)	42 (14.6)^b^
Lung Cancer History, N (%)	11 (3.8)
Instersticial Lung Disease, N (%)	4 (1.4)
Tumor Location, N, (%)	
Right Superior Lobe Left Superior Lobe Right Inferior Lobe Left Inferior Lobe Middle Lobe Lymph Node Right Central Left Central Trachea Bilateral	86 (30) 76 (26.5) 47 (16.4) 38 (13.2) 11 (3.8) 9 (3.1) 10 (3.5) 3 (1) 2 (0.7) 5 (1.7)
Total Lymph Nodes punctured, N (mean)	849 (2.95)
Number of Lymph Nodes punctured per EBUS, N (%)	
1 2 3 4 5 6	30 (10.5) 56 (19.5) 115 (40.1) 70 (24.4) 14 (4.9) 2 (0.7)
EBUS with ROSE, N (%)	130 (45.3)
Additional Biopsy, N (%)	
Not performed or not diagnostic Endobronchial Biopsie Transbronchial Biopsie	153 (53.3) 56 (19.5) 78 (27.2)
Major Surgical Procedure, N (%)	
Right Superior Lobectomy Left Superior Lobectomy Right Inferior Lobectomy Left Inferior Lobectomy Bilobectomy Right Pneumectomy Left Pneumectomy Segmentectomy or wedge resection Mediastinoscopy	71 (24.7) 51 (17.8) 23 (8.0) 23 (8.0) 12 (4.2) 27 (9.4) 22 (7.7) 48 (16.7) 4 (1.4)
NSCLC after surgical exploration, N (%)	238 (82.9 %)
Final Tumor Staging, N^c^ IA, N (%) IB, N (%) IIA, N (%) IIB, N (%) IIIA, N (%) IIIB, N (%) IV, N (%)	242 29 (12.0) 39 (16.1) 46 (19.0) 43 (17.8) 66 (27.3) 12 (5.0) 7 (2.9)

*COPD* chronic obstructive pulmonary disease, *Cardiovascular Disease* coronary arterial disease, arterial hypertension, ischemic or hemorrhagic brain disease, peripheral vascular disease, atrial fibrillation, aortic aneurysm, *EBUS* endobronchial ultrasound, *ROSE* rapid on-set evaluation, *NSCLC* non-small cell lung cancer

^a^smoking history could be assessed only when evidenced in the hospital discharge letter

^b^history of any cancer other than lung cancer

^c^considering 238 patients with NSCLC and 4 patients with Carcinoid Tumor

**Table 2:** EBUS-TBNA pathological findings

Absence of pathological findings, N (%)^a^	188 (65.5)
Squamous Cell Carcinoma, N (%)	40 (13.9)
Adenocarcinoma, N (%)	38 (13.2)
Large Cell carcinoma, N (%)	1 (0.3)
Adenosquamous Carcinoma, N (%)	1 (0.3)
NSCLC-NOS, N (%)	4 (1.3)
Clear Cell Carcinoma, N (%)	1 (0.3)
Undifferentiated Carcinoma, N (%)	2 (0.7)
Neuroendocrine Tumor, N (%)	1 (0.3)
Thymoma, N (%)	1 (0.3)
Lymphoproliferative Process, N (%)	2 (0.7)
Sarcomatous Process, N (%)	1 (0.3)
Anthracosis and Silica, N (%)	1 (0.3)
Granulomatous Process, N (%)	4 (1.4)
Inflammatory Process, N (%)	2 (0.7)
Total, N (%)	287 (100 %)

*NSCLC-NOS* non-small cell lung cancer not otherwise specified

^a^representative lymph node samples without disease

The EBUS-TBNA performance for the mediastinal staging of lung cancer was calculated by taking into account all 238 patients with NSCLC who had mediastinal lymph node sampling by both EBUS and surgery (Table 3). EBUS staging was correct in 213 cases and incorrect in 25. There were 180 true negative, 33 true positive, 21 false negative and 4 false positive findings. Those findings allowed us to calculate the NPV of 89 % (CI 95 % 84.5-93), the positive predictive value (PPV) of 89 % (72.5–95.7) the sensibility of 61 % (CI 95 % 47.8-72.9) and the specificity of 97 % (CI 95 % 94.5-99.1). From the 21 false negative cases, 16 (76 %) did not undergo positron emission tomography-computed tomography (PET-CT) or it was performed after the EBUS. In 15 (71 %) patients the affected lymph node chain was not punctured by EBUS-TBNA. Ten (47 %) patients had only lymph node metastases not directly accessible by the EBUS (lymph node chains 5,6,8 and 9). In 11 (52 %) patients the tumor was located in the left superior lobe. The more often affected lymph node chain was station 5, with 7 false negative cases.

**Table 3:** Mediastinal Lymph Node Staging: EBUS-TBNA X Surgery

EBUS-TBNA N stage	Final N stage	Number of cases
Correctly staged		
0 or 1	0 or 1	180
2	2	32
3	3	1
Incorrectly staged		
0 or 1	2	20
2	0 or 1	4
2	3	1

EBUS-TBNA: endobronchial ultrasound-guided transbronchial needle aspiration

Total of patientes with NSCLC: 238

True positive: 33 / True negative: 180 / False positive: 4 / False negative: 21

Sensibility: 61% (CI 95% 47.8-72.9) / Specificity 97% (CI 95% 94.5-99.1) / Positive predictive value 89% (CI 95% 75.2-95.7) / Negative predictive value: 89 (CI 95% 84.5-93)

Considering the 4 false positive cases, 2 were surgically classified as N1 and 2 were classified as N0 (all confirmed surgically as NSCLC). In just one of the cases there was a disagreement of the pathological findings of EBUS-TBNA and surgery (EBUS-TBNA suggested adenocarcinoma and surgery confirmed large cell carcinoma). All the 4 cases were submitted to surgical lobectomy and lymph node dissection.

Twenty patients had a mediastinoscopy after the EBUS (4 as the main surgical procedure and 16 as part of the mediastinal staging before surgery). There were 15 true negative, 1 true positive, 4 false negative and no false positive findings comparing mediastinoscopy to final surgical mediastinal staging. Mediastinoscopy did not contribute to a better mediastinal staging than the EBUS in any of the patients. All the 15 true negative cases were also negative by the EBUS. The true positive case was also positive by the EBUS, and one of the 4 false negative mediastinoscopies cases was positive by the EBUS.

## Discussion

Our study evaluated the EBUS-TBNA performance in a key subset of patients with lung cancer: those undergoing thoracic surgery. Despite not representing the majority of patients diagnosed with lung cancer, this is the subgroup in which we need to have the sharpest diagnostic and staging tools to ensure an accurate referral to surgery and expectation of cure. To our knowledge, this is the work with the highest number of patients submitted to surgery who had their EBUS-TBNA results directly compared with the surgical sampling. The use of EBUS was evaluated in a hospital routine, without adherence to study protocols influencing the exam.

The primary endpoint of the study was the proportion of patients with a final diagnosis of NSCLC by EBUS-TBNA correctly subtyped. Our results show that NSCLC was correctly subclassified in 79 % of cases. One of the limitations of existing evidence on EBUS diagnostic performance is that many studies included results of the index test and clinical follow-up in a reference standard test and did not account for the surgical sample being the only possible gold standard. This may have overestimated the result of some studies [13]. Esterbrook et al. showed that EBUS-TBNA samples when made into cell-blocks and subjected to a panel of immunohistochemical stains returned adequate tissue for NSCLC subtyping in 79 %, with a NSCLC-NOS reate of 21 % [14]. In a large, multicenter study, Navani et al. demonstrated that samples from EBUS-TBNA provide sufficient information for subtyping NSCLC in 77 % of the cases [15].

We chose the NPV of EBUS-TBNA for the mediastinal staging of lung cancer as the secondary endpoint of our study because we consider it to be the most clinically relevant measure in the subset of patients who undergo thoracic surgery. From a total of 238 patients with NSCLC surgically evaluated, 53 had mediastinal metastatic involvement ipsilateral to the target tumor lesion (N2 disease) and 2 had contralateral mediastinal involvement (N3 disease), representing a prevalence of mediastinal nodal involvement of 23 %. The results of our study showed an NPV of 89 %. Probably some of the most important publications of EBUS and mediastinal lymph node staging in patients with potentially resectable NSCLC are the ASTER trial [16], published in 2010, and the work of Yasufuku et al. [17], published in 2011. The ASTER trial found an NPV for endosonography staging alone without additional surgical staging of 85 % (prevalence of N2/N3 54 %). Yasufuku showed an NPV of 91 % for EBUS-TBNA (prevalence of N2/N3 disease 35 %).

The sensitivity of 61 % in our work was lower than expected and previously reported. This fact is due to the relatively high number of 21 false negative cases. In reviewing such cases we realize that 16 (76 %) did not undergo PET-CT or it was performed after EBUS and in 15 (71 %) the affected lymph node chain was not punctured by EBUS-TBNA. In the ASTER trial [16] all patients underwent PET-CT before EBUS. In the discussion of the Lung-BOOST trial [18], the authors suggest that a PET-CT may not be needed before EBUS-TBNA. In that trial EBUS-TBNA was performed using a systematic aspiration of all visible lymph node stations. Unfortunately, in our study, we are unable to determine if most of the procedures adopted a systematic or selective approach.

In 11 (52 %) patients from the false negative cases, the tumor was located in the left superior lobe and 10 (47 %) had only lymph node metastases not directly accessible by EBUS (lymph node stations 5, 6, 8 and 9). Endoscopic ultrasound (EUS), using the same scope as EBUS, was done in only 6 of our 287 cases. None of the false negative cases underwent also EUS in the same procedure as EBUS. The current consensus is that for a more complete needle-guided ultrasound evaluation of the mediastinum we should associate EBUS and EUS whenever necessary [19, 20]. Perhaps with the more frequent use of EUS in routine practice, some false negative results could have been avoided. However, even though recently showed to be feasible and safe [21], it would still be difficult to access the lymph nodes in chains 5 (the most often affected) and 6. This points to the need for more cautious strategies in patients susceptible to metastases in these chains, such as patients with tumors in the left superior lobe.

The 4 false positive cases represent patients with NSCLC. Unfortunately we do not have follow-up data, so is difficult to affirm that these are really false positive cases or maybe incorrectly surgically staged patients

The small number of 20 mediastinoscopies already reflects the lower use of this technique in our hospital routine. All mediastinoscopies were performed after EBUS to clarify questionable situations before the final surgical decision. Mediastinoscopy did not contribute to better mediastinal staging than EBUS-TBNA in any of those patients. These findings do not reflect the results of the ASTER trial [16] or corroborate the current recommendations of the main guidelines of American and European respiratory societies.

### Limitations

Some limitations apply to this study. First, it is a single-center study. We recognize that our results cannot be easily generalized. EBUS was performed in hospitalized patients under general anesthesia. Although this is a standard practice in our service, many procedures, probably the majority, performed in other bronchoscopy services around the world are performed in conscious or moderate sedation. Even tough the World Association for Bronchology and Interventional Pneumology (WABIP) guidelines [22] state that there is not enough evidence to recommend for or against any type of anesthesia, ideally, we should also have data in patients undergoing the procedure under conscious or moderate sedation. Second; two distinct pathology services have analyzed the samples obtained by EBUS-TBNA and surgery, which may have prevented results of one of the tests influencing the analysis of the other test. But it is difficult to clarify if the differences in pathological classification are due to the quality of the samples or a distinct interpretation by the pathologists. Third, at least 2 aspirates were routinely obtained from each target lesion per EBUS. Lee and colleagues [23] showed that, in the absence of ROSE, at least 3 aspirates should be obtained from each target lesion in order to provide optimal results from the test. Unfortunately we are not able to identify the number of aspirates that were used in each examination.

## Conclusions

In hospital routine and in the subgroup of patients eligible for surgical resection EBUS-TBNA has been proven to be a good tool for the primary diagnosis of lung cancer. The negative predictive value of 89 % for mediastinal staging of lung cancer is comparable to that reported in previous studies, but the relatively high number of false negative cases points to the need for standardization of routine strategies before, during and after EBUS.

## Abbreviations

CI, confidence interval; EBUS, endobronchial ultrasound; EBUS-TBNA, endobronchial ultrasound-guided transbronchial needle aspiration; EUS, endoscopic ultrasound; GIST, gastrointestinal stromal tumor; NPV, negative predictive value; NSCLC, non-small cell lung cancer; NSCLC-NOS, non-small cell lung cancer not otherwise specified; PET-CT, positron emission tomography-computed tomography; PPV, positive predictive value; ROSE, rapid on-site evaluation; WABIP, World Association for Bronchology and Interventional Pneumology
